# Abstracts and Gleanings

**Published:** 1878-01-20

**Authors:** 


### *ABSTRACTS AND GLEANINGS.*

### TREATMENT OF HYSTERIA.

John C. Peters, M.D., in a paper read before the Neurological Society, New York, remarks:

"Niemeyer says he accidentally hit upon a nervine of great efficacy in hysteria, and has made use of it with signal effect in many cases in which there were no indications for the local treatment of uterine disease, or where the hysteric symptoms persisted although the local uterine affection had been cured, viz: the *chloride of gold and sodium.* He has used it for several years and in a great number of cases, and was so encouraged by his success as to recommend it strongly to his pupils. It is well known to be a special re-agent upon the nerve tissues, much used in microscopical investigations. He prescribes five grains made into forty pills, with one drachm of gum tragacanth and a sufficient quantity of sugar; commencing with one pill after each meal, gradually increased, until eight are taken per day. But Dierbach, one of the best of the German writers on the *Materia Medica,* called attention to it over forty years ago. He describes it as exerting a most cheering influence upon the mind, and useful in many cases of debility, and even marasmus. It is said to excite the activities of the skin and kidneys, producing profuse and bad smelling perspiration, and discharges of urine, and an equally tonic and alterative effect upon the genital organs. As early as A. D., 980, Avicenna recommended it in various nervous affections, such as melancholy, hysteria, palpitations, asthmatic affections, etc. It was also used largely in the middle ages, by Raimond, Lull, Basil Valentine and Paracelsus; also by John Hartmann, the first professor of chemistry in Germany, Mynsicht and others. Finally, it was quacked with so much in the shape of so-called gold-pills, made of brick dust and madder, that its use was abandoned by reputable physicians. But Dr. Vogt says, after it has been taken for six or twelve days, in small doses, it will improve the appetite and general appearance; also, arouse more strength and activity in all the functions, especially in the brain and nervous system, causing greater cheerfulness and endurance. He recommends it in melancholia and hysteria. I have used it for thirty-five years, and am much pleased with it. Hahnemann got all his indications for its use from the above authorities.

*Cajeput oil* is a highly diffusible stimulant and antispasmodic, largely used in hysteria, flatulent colic, nervous vomiting, nervous dysphagia and nervous headaches. It is often rubbed on the back stomach and head in these affections, as well as given internally, and with considerable success.

*Cannabis indica* is largely relied upon in hysteria when coupled with exhausting menorrhagia and complicated with neuralgic headache, chorea, etc. I use it very frequently, and can recommend it.

*Chamomile oil* is an antidote to strychnia; it lowers reflex excitability in a remarkable manner; even that artificially excited by strychnia and brucia cannot only be calmed in a marked degree by it, but an animal fortified by this oil is not capable, according to Binz and Grisar, of being tetanized by a dose of strychnia which throws an unprotected frog of similar size into the characteristic spasms. It has been used successfully in hysterical cough; in spasmodic and pseudo-neuralgic persons; in pseudo-angina pectoris, and the colicky pains to which such persons are subject, and in hysterical pains in the fifth nerve.

*Cocculus indicus,* according to Phillips, is the great rival of nux vomica in nervous vomitings, when attended with a dull and heavy pain in the head, and intolerance of light and sound. Vomitings of this class are at times severe and persistent; at others there are alternations of constant nausea, with violent but ineffectual efforts to vomit. The stomach is often so exceedingly irritable that no description of food can be tolerated. Phillips says it seldom fails to give the relief so much desired, especially when the head is the part primarily affected, in patients who become nauseated from every disagreeable mental or moral emotion. It relieves the head first, and those of the stomach are soon after allayed. It also relieves the giddiness produced by derangement of the stomach; likewise the dyspepsia and constipation of nervous and hysterical subjects, especially when there is pain, nausea, giddiness and headache, with a feeling of hunger, attended with a repugnance to food, and when the colon is distended with a flatus, and the evacua ions more or less hard and lumpy. Phillips says coculus is often of singular efficacy in many of these cases. Also in tympanites, colic and other spasmodic affections of the stomach and bowels; in the nausea and colic of pregnancy; in dysmenorrhœa, and other painful affections of the uterus. It also rivals strychnia in the treatment of various forms of paralysis and paralytic stiffness of the limbs, with a loss of motor power in the legs, with giddiness and a feeling of lightness in the head. Phillips saw a patient recover in the course of a few weeks who had suffered for several months with loss of power in the legs—moving them with considerable difficulty, and who became giddy, nauseated and light-headed when he attempted to stand erect. He has seen well-marked cases of hysterical paralysis hysteric hemiplegia, choreic hemiplegia and epileptic hemiplegia successfully treated with cocculus, when the sensibility and muscular power were both much impaired.

Reil used it in paralysis of the sphincters, while Tschudi and Gubler resorted to *picrotoxine*, the active principle, in paralysis of the limbs and sphincters. The dose of the latter is about one-eighth to one-sixth of a grain. Picrotoxine acts on the motor centres, the vagus centres, and the centres for restraint of reflex movements; thus, convulsions can be excited by it in animals from which the brain has been removed. There is a general resemblance in its action to that of strychnine, for fish, under its influence, make twisting and boring movements; frogs get convulsions, or have semicircular swimming movements winding up with spasms, or singular movements of the legs. Sometimes tonic and clonic spasms alternate in the most singular but definite way, with swimming backwards and forwards, rolling over on the axis of the body, etc., etc. I have used cocculus for over thirty years, and frequently depend upon it and picrotoxine.

*Ignatia* has four times as much strychnia as nux vomica in it, and is, of course, a more powerful remedy. It is useful in many cases of hysteria, hypochondriasis and various nervous affections. Phillips says its good effects are considerable in globus hystericus, paralysis of the œsophagus, nervous aphonia, intercostal neuralgia, clavus hystericus, nervous hiccough, flatulent distention of the bowels, and especially in the convulsive bursts of crying and laughing and the general hyperæsthesia which attends hysteria. He claims to have rendered many hysterical cases much milder, and to have greatly prolonged the intervals between the attacks by the systematic use of two to five drops, or more, of the tincture several times a day. It is also claimed to rival gold in the treatment of melancholia, hypochondria, great mental depression, etc., and I can recommend it very highly.

*Lavender oil* is said to be more useful and agreeable in hysteria, nervous affections, headache, hypochondriasis, and flatulence than assafœtida.

*Orange-peel oil* was used by Imbert Gourbeyre for hysterical and other nervous affections, although he states that those who are exposed to its fumes long, are apt to be troubled with headache, dizziness, tinnitus, deafness, neuralgia, oppressed breathing, constriction of the throat, nausea, pyrosis, bad dreams, cramps and twichings of the muscles, and occasional convulsions. It is probably more useful in paralytic than in spasmodic cases.

*Sumbul* is said by Phillips to be a remedy for neuralgia of a certain type of more value than any other known drug. He says it is surprising to note the rapidity with which severe facial, sciatic, or ovarian neuralgia will yield to a few doses of sumbul, after resisting very powerful remedies. I have used it for ten years, more in nervous debility and dyspepsia than in neuralgia. It is a most admirable tonic and calmative."—*Virginia Medical Monthly.*

---

*AROMATIC SYRUP OF LIQUORICE.*

The following is a recipe for an aromatic syrup of liquorice which possesses such excellent qualities of disguising the taste of sulph. quinia that I think it my duty to communicate the formula to my pharmaceutical brethren :

Take of Pulverized Ext. of Liquorice ...4 ounces.
Jamaica Ginger, ................ .
Cinnamon Bark, ............ each 2 ounces.
Cloves, ........................ 1 ounce.
Sugar, .................... 60 troyounces.
Water, .............. a sufficient quantity.

Reduce the ginger, cinnamon and cloves to a coarse powder and boil in two pints of water over a slow fire for one hour. Then strain and dissolve in the liquid the pulverized extract of liquorice, with the aid of a gentle heat, stirring to assist the solution. When dissolved add the sugar, keeping up the heat till the latter is also dissolved. Then strain while hot and add hot water through the filter to make four pints of finished syrup.

The above syrup I find to disguise the taste of quinia better than syrup of liquorice root, the aromatic elixir of liquorice or the simple syrup of the extract of liquorice. It will completely cover the taste of 20 grains of quinia sulphate in one ounce of the syrup, and only a slightly bitter taste will be developed ten or fifteen minutes after taking, which, however, may be removed by taking a draught of black coffee with sugar.

This syrup has such a pleasant flavor, and none of the often objectionable sweetness of liquorice, that our physicians who are using it say that they find no trouble at all now in giving quinia in solution to children or to their most delicate patients. —*A Writer in Amerioan Journal of Pharmacy.*

---

*SANTONIN.*

Dr. Gibney, in New York Medical Association, inquired whether any unpleasant symptoms followed the use of five-grain doses of santonin for a child three years of age. Within the past year his attention had been called to poisonous effects produced by the drug given in five-grain doses to young children, since which time he had been guarded in its administration.

Dr. Porter replied that his usual method of administering santonin was to regulate the size of the dose by the age of the child, the number of grains corresponding to the number of years. In one case reported the child was three years old, and he used five-grain doses because it was thought there were indications for an active vermifuge. The case was one of supposed tape-worm, and no unpleasant effects were produced. The change which occurred in the color of the urine was sometimes alarming to the mothers, they supposing that their children were passing bloody urine. He, therefore, took occasion to allay their fears by forewarning regarding the change in color that might be expected.

[We have seen very distressing symptoms from the use of worm candy containg santonin, the patient, a four year old child, having taken a number of the lozenges.—Ed. Rec.]

---

*COMBINED USE OF CHLOROFORM AND MORPHIA.*

Prof. Konig, in a communication to the *Cbl. f. Chirurgie* (No. 39, 1877), says he has combined the hypodermic administration of morphia with that of chloroform in a large number of cases with very favorable results. It is seldom necessary to give more than one, or at most two centigrammes (1-6 to 1-3 gr.)

The indications for the use of morphia during chloroform-narcosis are twofold: 1. Motor disturbances occurring before or during chloroform-inhalation, unless these are very transitory. 2. Operations of such a nature that the chloroform-narcosis cannot be maintained throughout, and especially towards the end. Among the latter may be particularly mentioned operations upon the eye, plastic operations, extirpation of tumors from the soft parts of the face. The object of using morphia is to induce anæsthesia over and above the chloroform-narcosis, and also that this narcosis should not be pushed so far. As regards any danger which may be connected with the combination of narcotics, K. esteems this lightly. He says that out of some seven thousand cases in which he has used chloroform, none have died from it, and many of these took morphia also.—*Med. Times.*

---

### *CHLOROFORM HALLUCINATION AND ALLEGED RAPE.*

A surgeon was recently indicted at the Northampton Assizes (England) for rape, while having a tooth extracted. The offence charged was alleged by the prosecution to have been committed while the prosecutrix was so far under the effect of chloroform or chloric ether as to be speechless and motionless, but not unconscious. The defense set up was that the prosecutrix was under the influence of chloroform to such an extent as to have caused her to imagine that to have been done which she described. For the defense, Dr. B. W. Richardson was called, and stated from the evidence in the present case he should say that the patient had reached the second stage. The usual symptoms accompanying that stage were loss of consciousness and, in the case of women, development of any hysterical tendencies, any operation being impossible at this stage, the patient resisting and screaming if touched. It was at this period that the patient was subject to illusions. A person who was deprived of the power of motion by chloroform would, in his judgment, be deprived of the power of sight. He knew from his own personal experience that persons in the second stage were subject to delusions as to what had been done to them during the time. He gave an instance of a lady who, in the presence of himself, her father and mother, and a dentist's assistant, while under the influence of chloroform, brought a charge against the dentist who was operating upon her precisely similar to the one in the present case, and continued firm in the belief that the charge was well founded long after the influence of the chloroform had passed off, and probably still continued in that belief. The other medical witnesses who were called, and who expressed their concurrence in Dr. Richardson's evidence, were Mr. Mills, administrator of chloroform at St. Bartholomew's, Dr. Hawksley, Dr. Saundly, of Birmingham, and Mr. West, Senior Surgeon of Queen's College Hospital, Birmingham. It was stated that all the medical witnesses for the defense had come forward to give their evidence entirely gratuitously. The jury rendered a verdict of *Not Guilty.*

Moral—-Medical gentlemen should never administer anæthetics to a female unless in the presence of witnesses.—*New York Medical Record.*

---

### *THE EMPLOYMENT OF ANÆSTHETICS IN LABOR.*

M. Piachaud read a paper before the International Medical Congress of Geneva, in which he advanced the following conclusions:

1. The employment of anæsthetics is, as a general rule, advisable in natural labor.

2. The principal substances which have been used for this purpose up to the present time are ether, chloroform, amylene, laudanum, morphia hypodermically, chloral by the mouth and by injection.

3. Of these chloroform seems to be preferable.

4. It should be administered according to the method of Shaw, that is, in small doses at the beginning of each pain, its administration being suspended during the interval.

5. It should never be pushed to complete insensibility, but the patient should be held in a state of semi-anæsthesia, so as to produce a diminution of the suffering.

6. The general rule is never to administer chloroform except during the period of expulsion; but in certain cases of nervousness and extreme agitation it is advantageous not to wait for the complete dilatation of the os.

7. Experience has shown that anæsthetics do not arrest the contractions of the uterus or abdominal muscles, but that they weaken the natural resistance of the perineal muscles.

8. The use of anæsthetics has no unpleasant effect on the mind of the mother or upon the child.

9. In lessening the suffering, anæsthetics render a great service to those women who dread the pain; they diminish the chances of the nervous crises which are caused during labor by the excess of suffering; they make the recovery more rapid.

10. They are specially useful to calm the great agitation and cerebral excitement which labor often produces in very nervous women.

11. Their employment is indicated in natural cases until the pains are suspended or retarded by the suffering caused by maladies occurring previous to or during labor, and in those cases where irregular and partial contractions occasion internal and sometimes continuous pain, without causing progress of the labor.

12. In a natural labor, chloroform should never be used without the consent of the woman and her family.

M. Courty advocates the use of chloroform. He thinks it indicated when the pains are very great and irregular, or where the patient demands it.

M. Leblond prefers to use the hydrate of chloral.— *Gazette Medicale, Oct.* 20, 1877.

[We have used chloroform in all conditions of the parturient woman. In rigid os or perineum, in excessive nervousness or extreme sensibility to pain, it is wise to use "it" in the manner above described; but in favorable and uncomplicated cases, we now seldom use it, because it predisposes to hemorrhage immediately after delivery, while its secondary effects are restraining to the lachral discharge, tends to the accumulation of clots, and increased after-pains, and retards the lachral secretion. When given, it is well to give a dose of ergot immediately after delivery to guard against hemorrhage, and, after a few hours, moderate doses of gelseminum to encourage the lachral discharge.—W. Ed.]

### *THE TREATMENT OF OZÆNA.*

Dr. Rouge, in a paper read at the third meeting of Congress, arrived at the following conclusions:

1. Ozæna is the result of suppuration in the nasal fossæ or their annexes—viz., the frontal, maxillary, and sphenoidal sinuses, and the ethmoidal cells.

2. The suppuration appears to originate in all cases in disease of the nasal fossæ or their annexes.

3. The degree of fœtor of the air issuing from the nasal fossæ depends on the extent of the osseous lesions which have given rise to the ozœna.

4. The latter is also increased by the stagnation of pus in the sinuses.

5. Should the surgeon fail to trace the disease to some affection of the nasal cavity, he must seek for it in the sinuses and the ethmoidal cells.

6. The local treatment of ozæna comprises:

(*a.*) Frequent washing out of the nasal cavity by means of injections, which vary with the spinal indications of each case. (*b.*) The insufflation of disinfecting, caustic, or astringent powders. (*c.*) Cauterization of various kinds: the use of the galvano-cautery. (*d.*) In severe cases all sequestra must be removed, and the sinuses completely drained. The nose is detached by the sub-labial method; this enables us to explore the nasal fossæ directly, to remove all necrosed portions of bone, and to open the sinuses. The formation of a cicatrix is thus avoided.

7. It is unnecessary to speak of any general mode of treatment, as this must be regulated according to the patient's constitution.

M. Verneuil said he had not applied the method of Dr. Rouge, but he would do so. He remembers that M. Trelat had some good results from it.

Dr. Ollier adopted this method in certain cases.

Dr. Rouge believed that ozæna is genally due to alteration of the sinuses, but more frequently to caries of the ethmoidal cells. Posterior rhinoscopy is not so useful, because the mucous membrane is so swollen that we cannot conveniently discover the diseased parts.—*Med. Press and Circ.*, Oct. 10, 1877.

---

### *ULCERATION OF THE OS UTERI.*

*General Treatment.*—We can not too forcibly inculate the necessity of absolute rest in the horizontal position. By this means, congestion about the uterus is lessened, and the ulcerated surface prevented from impinging on any part. The diet should be liberal. The bowels should be kept well opened. All marital intercourse should be forbidden.

*Medicine.* — There being generally a state of anæmia to contend against, we would first recommed the vegetable tonics and cod liver oil, afterward the ferruginous preparations. Where any induration exists, iodide of potassium should be administered. It is essential to raise the tone of the body, as concurrently with its improvement, so the healing process will be expedited.

*Topical Applications.*—Much care is required in deciding whether to deplete or not, in chosing the form of caustic to be applied, and in prescribing an effectual injection. In all cases where the veins are prominent about the os, we would commence either by leeching or puncturing with the lancet. The latter we prefer. In cases of slight ulceration, touching the part with nitrate of silver or chromic acid, followed by a plug of cotton-wool steeped in glycerine, is generally effectual. Should the ulceration be obstinate, we would apply fuming nitric acid. The cotton-wool, saturated with glycerine, must be introduced daily. Where the lips of the os are divided, it must be concluded that the inflammation has extended along the cervical canal. In these cases the external os should be well burned with the caustics named; if necessary, the actual cautery should be employed; but the cervical canal must not be molested. These failing, plugs of iodized cotion-wool should be applied daily.—*Phil. Med. and Surg. Reporter.*

---

### *PROTRACTED LABOR.*

On this subject, E. Staumore Bishop, in *London Med. Ex.*, remarks:

"I said that the child's head was the natural stimulus to the maternal vaginal fibres. As it descends it involves more and more peripheral ends of nerves in its pressure; reflex currents are excited, and the uterus contracts more and more strongly. Can we imitate this? I think we can. If you pass two fingers of the right hand into the vagina, and place the tips slightly divergent upon the posterior wall, wait for a pain, and, when it begins, slowly and with measured force make gradually descending pressure upon the rectum, passing downward over the perinæum, and so to the vulva. As the pain abates, gradually take off your pressure, and during the interval do not press at all. In this way you cheat the uterus, you cheat the patient into acting as though the child's head were lower than it really is. Members may smile, but I can assure them that over and over again, by adopting this expedient, I have found the nervous cry and the useless shrink of these nervous patients disappear, and, instead of drawing back and as of set purpose deliberately thwarting the natural efforts, the patient has settled down to her work and been saved from forceps. I firmly believe that in this way the forceps have often been rendered unnecessary, where but for this plan the patient would have exhausted herself, and the use of instruments would have been unavoidable."

---

### *SUBSTITUTES FOR QUINIA.*

Prof. Theo. Husemann, in the course of a long article, in which he discusses the relative value of those remedies which have at various times been proposed as substitutes for quinia, draws attention to an alkaloid, which alone can be regarded as a rival of quinia, standing in effectiveness between it and other cinchona alkaloids, and which has fallen in o undeserved oblivion. This alkaloid is *Bebeeria* (or Bibiria, according to Binz), obtained from beebeeru-bark, which is also used as dye-wood, and is derived from *Nectandra Rodiœi* Schomburgh, a tree belonging to the natural order Lauracæ, and native of Guiana. From the investigations of Binz and Conzen, it appears that the physiological effect of bibeeria is identical with that of quinia.—*Pharm. Handel.*

---

### *THE TREATMENT OF SCIATICA.*

Dr. Flemming gives, in the Berlin *Klinisthe Wochenschrift,* the result of his experience of forty cases of sciatica by means of the sand-bath. The patient is placed in a kind of trough, and the affected limb is surrounded by sand, at a temperature of 100° Fahr. or more, for half an hour; after this, a warm water bath is administered. Recovery is stated to take place, upon the average, after twenty-four sand baths.—*London Lancet.*

---

### *EXTRACTION OF QUINAMIA FROM RED BARK.*

The alkaloid quinamia was discovered by Hesse, in 1872, in the bark of *Cinchona succirubra,* cultivated at Darjeeling, in British Sikkim. It contains one molecule of water more than cinchonia or cinchonidia, and differs from quinia and quinidia by having two more atoms of hydrogen.—*L' Union Pharm.*

---

### *THE POISONOUS DOSE OF CASTOR OIL SEEDS.*

It has long been known that the seeds of *Ricinus communis* contain, besides oil, a peculiar acrid principle, which causes violent vomiting and purging, and which must be reckoned among the acrid poisons. Van Hasselts, a number of years ago, declared one seed to be sufficient to sicken a grown person, and twenty to be sufficient to kill him.—*New Rem.*

### *ANTAGONISM OF BELLADONNA AND OPIUM.*

The recent Edinburgh Committee, presided over by the lamented Dr. J. Hughes Bennett, concluded—

1. That sulphate of atropia is, within a limited range, physiologically antagonistic to meconate of morphia.

2. Meconate of morphia does not act antidotally after a large dose of atropia; thus, while atropia is an antidote to morphia, morphia is not an antidote to atropia.

3. Meconate of morphia does not antagonize the effect of atropia on the branches of the vagi supplied to the heart.

Both agents in question destroy life in the same manner. Opium is more antagonistic to belladonna, than belladonna is to opium. In opium poisoning, treated by belladonna, it should be suspended so soon as the pupils begins to dilate, for after this it intensifies the action of the opium. In the majority of reported cases of opium poisoning, treated by belladonna, the reporter acknowledges that either copious emesis, free stimulation, or faradism, had been resorted to along with belladonna.—*Dr. Milliken in Med. Asso. of Ohio.—Ohio Med. and Surg. Jour.*

---

### *COFFEE AS AN ANTIDOTE TO STRYCHNIA.*

Dr. Attilio Lelli having met with a case in which a large dose of strychnia was administered in coffee without fatal consequences, was led to institute some experiments to determine whether it possessed an antitoxic power against this drug. The animals employed were rabbits, and by comparative trials he found that a dose of five centigrammes proved fatal in a short space of time; when the same or a larger dose was given in a very strong infusion of coffee, he found that the coffee either acted as a complete antidote in preventing the poisonous effects of the strychnia, or that it materially diminished the violence of its action. The details of the experiments are given in the *Rivista Sperimentale di Freniatria,* edited by Prof. Carlo Livi, of which the first Fasciculus of the third volume has just been issued.—*London Lancet.*

*THE USE OF THE TREPHINE IN DEPRESSSED FRACTURES OF THE SKULL.*

(*The British Medical Journal*, July 21, 1877).—Dr. Robert S. Hudson, after alluding to the change in surgical opinion which has occurred since the time of Pott, and to the brilliant results which that surgeon obtained by the use of the trephine, proceeds to question the propriety of that change, and asks that the surgical practice of the mining districts around Cornwall be given its due weight in the consideration of the question. For many years the operation of trephining for depressed fracture of the skull has been of weekly, almost daily, occurrence, and, according to Dr. Hudson, a very large percentage of the cases recover. If death ensue, there are generally obvious causes to account for it, such as diffused injury with laceration of brain-substance, and fractured base; success usually depends on an early operation, as soon as possible after the accident. He sums up his remarks as follows:

"1. Surgeons practicing in the mining districts around Redruth and Camborne have had, especially in former times, usually opportunities for the study ol head-injuries.

"2. In compound fractures of the cranium, it has been the invariable practice of the most experienced to elevate depressed bone by means of the trephine or Hey's saw, without waiting for symptoms of compression or irritation.

"3. It is believed by those surgeons that no danger whatever attaches to the operation *per se;* pyæmic risks are unknown; and recovery is the rule after trephining operations.

"4. So firm is popular belief in the efficacy of the trephine, that a surgeon who hesitated to employ it, under the plea of waiting for symptoms, would assuredly suffer in reputation, if, in the event of death, he were not put on his trial for manslaughter.

"5. Hospital statistics place herniotomy among the most dangerous operations; but the statistics of hospital surgeons in their private practice show to a demonstration that an operation for the reduction of strangulated hernia is practically harmless, even when it is necessary to open the peritoneal sac, and that the risk is directly proportionate to the length of the ignorant delay which has been allowed to exist previous to the operation. (Holme's System of Surgery, vol. iv. page 692.) Although the parallel is not in every respect a complete one, we employ the trephine at the earliest possible period, and aim at preventing mischief by removing all sources of irritation.

"6. No matter how deeply prejudiced against the trephine our young surgeons may be when fresh from the schools, a few years' experience generally dispels the illusion; they become converts to the practice of the districts, and cease to look on its employment as antiquated surgery."

In *Guy's Hospital Reports* for 1877, Mr. Davies-Colley contributes two interesting cases in which the trephine was successfully employed, and adds, "These two cases support the rule which most of our text-books either miss or fail to impress, that in punctured fracture of the skull it is the surgeon's duty to trephine at once, without waiting for symptoms of compression or irritation."—*Med. Times.*

---

*WASHING OUT THE BLADDER.*

Dr. H. H. Hill, in the *American Medical Weekly*, reports the following case:

Mr. L., age eighty, an estimable and highly respectable gentleman, had up to the 28th of August, 1875, been a sufferer from chronic disease of the bladder (cystic) of ten or twelve years' duration, the disease and suffering gradually increasing until he was confined to his house, and finally to the bed He had "put his house in order," and made all preparations for the final end, which seemed near. He often asked my advice as to what he could or should do for himself, and I as often advised and prescribed for him with no permanent relief; saying to him, so long as you can keep the bladder empty I will not interfere with the catheter; an operation I much dreaded, as he often had hemorrhage, sometimes profuse, and also a profuse muco-purulent discharge with the urine, and very offensive.

August 28, 1875, he could no longer free the bladder; his suffering rapidly increasing, I passed a catheter without much difficulty, and drew off about a pint and a half of urine and offensive matter, which gave him great relief. I repeated the operation every eight hours. The general condition of my patient was in every way bad; very much emaciated; unable to take any kind of nourishment, except a little milk; the sight or smell of any other kind of food would cause him to vomit; vomiting was frequent; chills were severe, with profuse sweats.

On examination, over the region of the bladder, I found an oval tumor extending from the pubes to within one inch and a half of the umbilicus, and equally broad. The prostate gland was enlarged to the size of a hen's egg. The condition of my patient was such that I did not expect him ever to get any permanent relief. After visiting him a few days, I proposed to him washing the bladder with warm water, in order to get rid of some of the stench. This operation I did thoroughly twice every twenty-four hours with a four-ounce syringe, passing the water through the catheter, and taking it back into the syringe, empty it, and repeat the operation four, five and six times, or until the water came back clean. The washing afforded him relief daily, while his general health began to improve gradually. Vomiting, sweating and chills soon left him; appetite returned gradually, and at the end of two months he was able to be up and perform the operation of washing his own bladder by the aid of a fountain syringe. The prostate gland is now of normal size, and no tumor in the region of the bladder. Mr. L. has always been a very active merchant, and still is for a man of his years. His weight now is about 180 pounds. He still continues his washing.

Now, Doctor, I do not report the above case as anything new by way of washing the bladder, but I am of the opinion that we have been in the habit of neglecting that organ entirely too much as to local treatment. Since treating the above case, I have been very liberal in the use of warm water in the bladder, and in no instance without perfect satisfaction. Soft water 98 to 100° F. I never use a double catheter, as I consider quantity and moderate distension important.—*Amer. Med. Weekly.*

---

### *CONIUM.*

As an agent affecting the circulation in the brain and spinal cord, and as a paralyzant of voluntary muscles by its effect upon the afferent spinal nerves, conium has not yet attained the high place in general practice which it deserves. Perhaps the two circumstances which have led to this are, first, that in diseases requiring the use of conium, as in spinal irritation, congestion, meningitis, etc., the medicine is seldom employed in sufficient dose; second, there is very little of the drug which is reliable. Conium should be administered as digitalis; *i. e.*, for its effects alone, without reference to quantity. Dr. Harley has declared that conium is to the corpora striata, the smaller nerve centers, and the entire motor tract, "what opium is to the brain." Since I have been less careful in regard to the dose, I have had better results from the use of conium. Some years ago I made extensive use of extract of conium in cerebro-spinal meningitis, and with marked benefit. The only preparation which is at all reliable is the fluid extract.—*Cin. Med. News.*

---

### *SALICYLIC ACID.*

The effect of this acid in controlling acute rheumatism is truly wonderful. Much of its value no doubt depends upon the sedation exerted by it upon the circulation, as a consequence of which pain is lessened and temperature reduced. I have found the pain of migraine and other neuralgias yield very promptly to its use. As a local application to the nasal and pharyngeal mucous membrane in diptheria and other diseases it is unexcelled. Its caustic nature demands care in its use, especially in young children, and the following formula makes an excellent and safe mode for its administration:

R.—Acidi salicylic....................dr. iss.
Ammoniæ citratis..............dr. ss ad dr. i.
Syrupi cinnamoni....................oz. iss.
Aquæ cinnamoni.....................oz. iss.

M. Ft. Teaspoonful every second hour for a child of five years suffering with rheumatism.

The putrescent character of the stools in children suffering with summer diarrhœas is at once changed by salicylic acid, and a corresponding improvement in the condition of the little patient noticed. Its power over living germs renders it at once invaluable when contagion is feared. Prof. Abelin, of Stockholm, says that "in children, doses large enough to bring down temperature acted as a poison," and cites a case in which twelve grains caused death. In such doses it seemed to be a corrossive poison. In smaller quantities it lowers temperature without exerting any beneficial effect upon the course of the disease.—*Cin. Med. News.*

### *CHLORAL HYDRATE.*

It must not be forgotten that the symptoms relieved by chloral hydrate and potassium bromide are dependent upon hyperæmia of the nerve centers in the brain or cord, and that sudden exhaustion is attendant upon many diseases of infants; *e. g.* cholera, diarrhœa, etc., in which convulsions usually terminate life. Chloral and bromide would but increase the trouble, and stimulants alone are indicated. The apyretic action of chloral hydrate renders the mixture additionally valuable in high temperature when convulsions threaten.

The local use of hydrate chloral is scarcely less valuable. I now depend upon its prompt and pleasant action in diptheria; to abort absesses, and to prevent the formation of pus in sinuses, as a gargle in stomatitis and in scorbutic gums of childhood, it is unexcelled, as well as in the angina of eruptive fevers. Chloral hydrate and bromide of potassium are contra-indicated in chorea. The rapid anæmia in these cases is of itself sufficient reason to predict what practice confirms. In whooping-cough a combination of the bromides, as in the formula of Dr. Brown-Sequard, will, if pushed, always give satisfaction. As a general thing in such cases the doses are far too small, and the interval too long.—*Ib.*

### *ERGOT.*

Ergot produces vaso-motor spasm, and consequently increased arterial tension, through its action upon the nerve centers within the craneum. This fact, if it be conceded, gives to the drug a therapeutic importance, in treatment of diseases affecting the circulation, perhaps unequaled by any other medicine. I have made extensive use of ergot based upon the above theory, and so far with the best results. The importance of ergot as a therapeutic agent in congestions of the brain and spinal cord in childhood, in catarrhal and mucous diseases, etc., renders it especially proper to include it in the medicines of childhood.—*Ex.*

### *IODINE PURPURA.*

The *Revue Mensuelle de Medicine et de Chirurgie* contains a description, by Fournier, of a form of purpura, which results from the administration of iodine, three cases of which he had carefully observed. This iodine purpura has its seat upon the anterior surface of the thighs, and consists of scattered miliary petechiæ; it is unaccompanied by general symptoms. It appears a few days after beginning the administration of iodine, becomes more marked as the dose is increased, and disappears upon the discontinuance of the medicine. Upon recommencing the iodine treatment he was always able to cause a return of the purpura.—*Allg. Med. Central Zeitung,* No. 88, 1877.

### PRURITUS VULVÆ.

Among the best remedies we have ever found for this troublesome affection is the following:

R—Acidi carbolici.............................. gtt. x
Glycerin
Water.......................................aa – oz. j

M. Apply locally.—*Zeimsen.*

